# Hypoxia- and Postirradiation reoxygenation-induced HMHA1/ARHGAP45 expression contributes to cancer cell invasion in a HIF-dependent manner

**DOI:** 10.1038/s41416-024-02691-x

**Published:** 2024-05-13

**Authors:** Peter W. T. Lee, Tatsuya Suwa, Minoru Kobayashi, Hui Yang, Lina R. Koseki, Satoshi Takeuchi, Christalle C. T. Chow, Takaaki Yasuhara, Hiroshi Harada

**Affiliations:** 1 Laboratory of Cancer Cell Biology, Graduate School of Biostudies, Kyoto University, Kyoto, 606-8501 Japan; 2 Department of Genome Repair Dynamics, Radiation Biology Center, Graduate School of Biostudies, Kyoto University, Kyoto, 606-8501 Japan; 3 Department of Oncology, University of Oxford, Oxford, OX3 7DQ UK; 4 Laboratory of Genome Stress Response, Graduate School of Biostudies, Kyoto University, Kyoto, 606-8501 Japan; 5 Department of Late Effects Studies, Radiation Biology Center, Graduate School of Biostudies, Kyoto University, Kyoto, 606-8501 Japan

**Keywords:** Radiotherapy, Cancer microenvironment

## Abstract

**Background:**

Cancer cells in severely hypoxic regions have been reported to invade towards tumour blood vessels after surviving radiotherapy in a postirradiation reoxygenation- and hypoxia-inducible factor (HIF)-dependent manner and cause recurrence. However, how HIF induces invasiveness of irradiated and reoxygenated cancer cells remains unclear.

**Methods:**

Here, we identified human minor histocompatibility antigen 1 (HMHA1), which has been suggested to function in cytoskeleton dynamics and cellular motility, as a responsible factor and elucidated its mechanism of action using molecular and cellular biology techniques.

**Results:**

HMHA1 expression was found to be induced at the transcription initiation level in a HIF-dependent manner under hypoxia. Boyden chamber invasion assay revealed that the induction of HMHA1 expression is required for the increase in invasion of hypoxic cancer cells. Reoxygenation treatment after ionising radiation in vitro that mimics dynamic changes of a microenvironment in hypoxic regions of tumour tissues after radiation therapy further enhanced HMHA1 expression and invasive potential of HMHA1 wildtype cancer cells in ROS- and HIF-dependent manners, but not of HMHA1 knockout cells.

**Conclusion:**

These results together provide insights into a potential molecular mechanism of the acquisition of invasiveness by hypoxic cancer cells after radiotherapy *via* the activation of the ROS/HIF/HMHA1 axis.

## Introduction

Intratumoral hypoxia commonly occurs in solid tumours [1, 2] and there has been an increasing amount of research showing that cancer cells acquire malignant properties like invasiveness and therapy resistance under hypoxic conditions, linking to poor prognosis in patients [3–6].

Hypoxia-inducible factor (HIF), the master regulator of metazoan hypoxic responses, functions as a heterodimeric transcription factor composed of an α-subunit, HIF-1α, HIF-2α, or HIF-3α, and a β-subunit, HIF-1β/ARNT (aryl hydrocarbon receptor nuclear translocator), and governs the expressions of a myriad of downstream genes [7–9]. The activity of HIF is mainly regulated at posttranslational levels through the stabilisation of the α-subunits [10–12]. For example, under normoxic conditions, HIF-1α is hydroxylated by the O_2_/Fe^2+^/2-oxoglutarate-dependent dioxygenase (2-OGDD), prolyl-4-hydroxylase domain protein (PHD) [13–15], and is then recognised by the von Hippel–Lindau protein (pVHL)-containing E3 ubiquitin ligase, resulting in the proteasome-mediated degradation of HIF-1α protein [10, 11, 16, 17]. In addition, factor-inhibiting HIF-1 (FIH-1), which is another 2-OGDD, hydroxylates HIF-1α at its C-terminal transactivation domain, thereby suppressing its transactivation activity under normoxia [18, 19]. Contrarily, under hypoxic conditions, the lack of oxygen inactivates the 2-OGDDs, and HIF-1α-subunit is stabilised. It then interacts with the constitutively expressed HIF-1β protein, and is recruited to its recognition sequence, hypoxia-response element (HRE) to induce the transcription of downstream genes [7, 8, 20–22].

Metastasis, the major cause of mortality in cancer patients, comprises a series of biological events, starting from local invasion, intravasation and extravasation, to micrometastasis formation and colonisation at distant organs [23, 24]. Previous studies have shown that hypoxic stimuli and HIF contribute to each step of the metastatic cascade, and there have been accumulating data on the molecular details of hypoxia-induced metastasis [25–28]. In parallel, clinical analyses have also revealed that HIF-1α expression is associated with higher metastasis incidence and/or poor prognosis in breast, colon, nasopharyngeal cancer, and soft tissue sarcoma patients [29–33].

Anticancer treatments, such as radiation therapy, have been suggested to stimulate cancer invasion and metastasis [34, 35]; however, the molecular bases behind them still remain elusive. Previously, we reported that severely hypoxic cancer cells lying approximately 100 μm away from tumour blood vessels experience reoxygenation after surviving radiation therapy, and subsequently translocate towards tumour blood vessels in a HIF-1-dependent manner [36, 37]. Nonetheless, the molecular mechanisms underlying both the stimulatory effect of hypoxia as well as postirradiation reoxygenation treatment on the invasiveness of cancer cells remain largely unknown.

In the present study, we demonstrated that human minor histocompatibility antigen 1 (HMHA1; also known as ARHGAP45) is induced under hypoxic conditions in a HIF-dependent manner, and is responsible for the augmented invasion activity of hypoxic cancer cells. Moreover, we showed that irradiation followed by reoxygenation induces HMHA1 expression *via* the ROS-HIF axis, and HMHA1 is necessary to the enhanced cancer cell invasion after irradiation and reoxygenation.

## Results

### HMHA1 expression is induced under severely hypoxic conditions

**Fig. 1:**
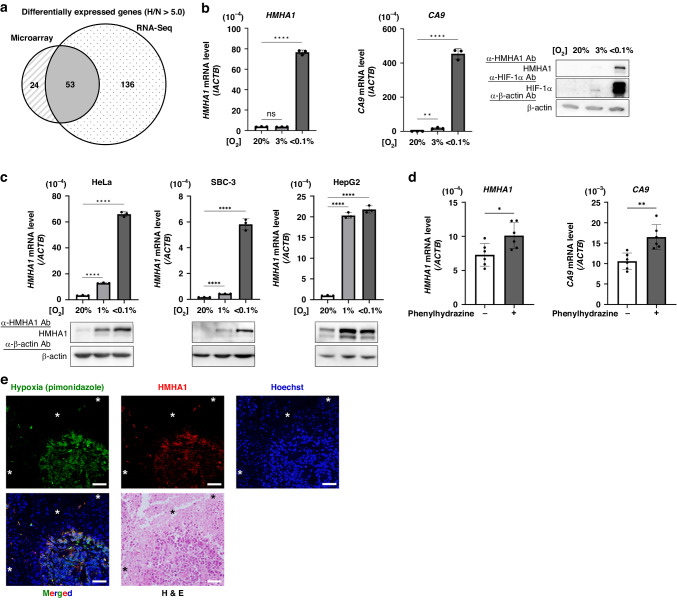
HMHA1 expression is induced under severely hypoxic conditions. **a** HeLa cells were cultured at normoxic (N, 20% O_2_) or severely hypoxic (H, < 0.1% O_2_) condition for 24 h, followed by genome-wide DNA microarray and RNA sequencing (RNA-Seq) analyses. Differentially expressed genes with induction ratio > 5.0 were identified and the Venn diagram shows the overlapping candidate genes. **b**, **c** qPCR (for *HMHA1* and *CA9* mRNA in (**b**) and *HMHA1* mRNA in (**c**) and Western blotting (for the indicated proteins) using HeLa cells (**b**) and the indicated cells (**c**) cultured under the indicated oxygen conditions for 24 h. Results are shown as mean ± s.d. (*n* = 3); ns: not significant, **P < 0.01, ****P < 0.0001 (Student’s *t*-test). **d** qPCR (for *HMHA1* and *CA9* mRNA) using HeLa tumour xenografts from untreated or phenylhydrazine-treated mice. Results are shown as mean ± s.d. (*n* = 6); *P < 0.05, **P < 0.01 (Student’s *t*-test). **e** FFPE sections of HeLa tumour xenografts were double-stained with antibodies against the hypoxia marker, pimonidazole (green), and HMHA1 (red). Nucleus was counterstained with Hoechst 33342 (blue). Scale bar: 50 µm. *: blood vessel. Reproducibility was confirmed in xenografted tumours from three independent mice and representative images are shown.

Hypoxia has been shown to confer malignant properties, like invasiveness, on cancer cells. To elucidate the underlying molecular mechanism, we aimed to explore a hypoxia-responsive and invasion-related gene. We previously performed a genome-wide microarray analysis to compare gene expression profiles of cells cultured under normoxic condition and that under severely hypoxic condition and deposited the resulting dataset in the Gene Expression Omnibus (GEO) database at the National Center for Biotechnology Information (NCBI) (accession number GSE161393) [38]. Here, we additionally performed RNA-Seq analysis to independently extract hypoxia-inducible genes (GEO accession number: GSE254480). With a cut-off value of 5.0 for the hypoxia/normoxia induction ratio, 77 and 189 genes exhibited hypoxia-responsive expression in the microarray and RNA-Seq analyses, respectively (Fig. 1a; Supplementary Table S1 and S2). Fifty-three of these genes were commonly induced under hypoxia in the two experiments, and thus were subjected to an analysis using the Gene Ontology Annotation (GOA) Database (https://www.ebi.ac.uk/GOA/) to scrutinise their biological functions (Supplementary Table S3). Unexpectedly, apart from *LOXL2* which has been well studied in relation to EMT and metastatic niche formation [39–41], none were annotated with cell motility-related terms amongst the 53 candidate genes. However, we decided to focus on the *HMHA1* gene here since it has been reported to be associated with migration and invasion of cancer cell [42] in addition to with both actin cytoskeleton dynamics of various cells [43] and migration of naïve T cell [44], and moreover since its hypoxia-inducible property has not been reported.

To start with, we investigated the effect of hypoxia on HMHA1 expression by culturing HeLa cells in various oxygen concentrations, and assessed the expression levels of *HMHA1* mRNA by RT-qPCR. Whilst the expression of carbonic anhydrase 9 (*CA9*), a well-known hypoxia-inducible gene, significantly increased under both mildly hypoxic (3% O_2_) and more severely hypoxic (1% and < 0.1% O_2_) conditions, the induction of *HMHA1* occurred only under severe hypoxia (Fig. 1b). Similarly, Western blot analysis also showed that the protein levels of HMHA1 increased only when cells were cultured under severely hypoxic conditions (Fig. 1b). Next, we employed a panel of other human cancer cell lines to examine whether the hypoxic induction of HMHA1 expression is a common phenomenon in other cancer cells besides HeLa. Under hypoxic conditions, the mRNA levels of *HMHA1* increased, albeit to different extents, in all tested cell lines, showing that HMHA1 expression is indeed induced by hypoxia in cancer cells (Fig. 1c; Supplementary Fig. S1A).

In order to test whether the hypoxic induction of HMHA1 also arises in vivo, we treated mice bearing subcutaneous HeLa tumour xenografts with a haemolytic reagent, phenylhydrazine, and pharmaceutically depleted oxygen supply to the tumour tissue. Alongside the increase in kidney erythropoietin (*Epo*) mRNA expression, which denotes the physiological response to acute anaemia, mRNA levels of *HMHA1* and *CA9* in the xenografted tumours were also elevated in the phenylhydrazine-treated mice, indicating that low oxygen availability can upregulate HMHA1 expression in tumours (Fig. 1d, Supplementary Fig. S1B). To investigate the relationship between intratumoral hypoxia and HMHA1 expression more directly, we prepared another group of tumour-bearing mice and injected pimonidazole, a hypoxia marker, into them before tumour excision. Serial sections of the formalin-fixed paraffin-embedded tumours were subjected to immunohistochemical analyses. Juxtaposing H&E-stained section with that stained with anti-pimonidazole antibody, we detected hypoxic regions distant from blood vessels in the tumours. Moreover, immunostaining using anti-HMHA1 antibody detected the expression of HMHA1 in pimonidazole-positive hypoxic regions (Fig. 1e, Supplementary Fig. S1C). Taken together, these results support that HMHA1 expression is indeed induced in the hypoxic regions of solid tumours.

### HMHA1 expression is regulated at the transcription initiation level in a HIF-dependent manner

**Fig. 2:**
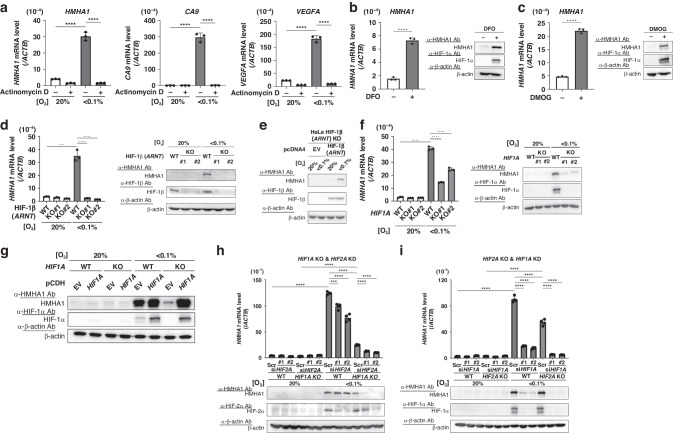
Hypoxic induction of HMHA1 is HIF-dependent. **a** qPCR (for *HMHA1*, *CA9*, and *VEGFA* mRNAs) using HeLa cells cultured with or without actinomycin-D treatment (10 μg/mL) under the indicated oxygen conditions for 24 h. Results are shown as mean ± s.d. (*n* = 3); ****P < 0.0001 (Student’s *t*-test). **b**, **c** qPCR (for *HMHA1* mRNA) and Western blotting (for the indicated proteins) using HeLa cells with or without deferoxamine (DFO, 100 μM) (**b**) or dimethyloxalylglycine (DMOG, 2 mM) (**c**) treatment for 24 h at 20% O_2_. Results are shown as mean ± s.d. (*n* = 3); ****P < 0.0001 (Student’s *t* test). **d** qPCR (for *HMHA1* mRNA) and Western blotting (for the indicated proteins) using parent HeLa cells (WT) or HIF-1β knockout clones (KO#1 and #2) exposed to the indicated oxygen conditions for 24 h. Results are shown as mean ± s.d. (*n* = 3); ***P < 0.001, ****P < 0.0001 (Student’s *t* test). **e** HIF-1β knockout HeLa cells transiently transfected with HIF-1β expression vector or its corresponding empty vector (EV) were cultured under the indicated oxygen conditions for 24 h and subjected to Western blotting (for the indicated proteins). **f** Same experiments as in (**d**) were performed using HIF-1α knockout clones. Results are shown as mean ± s.d. (*n* = 3); ****P < 0.0001 (Student’s *t* test). **g** Parent HeLa cells (WT) or HIF-1α knockout HeLa cells stably transfected with HIF-1α expression vector or its corresponding empty vector (EV) were cultured under the indicated oxygen conditions for 24 h and subjected to Western blotting for the indicated proteins. **h** qPCR (for *HMHA1* mRNA) and Western blotting (for the indicated proteins) using parent HeLa cells (WT) and HIF-1α knockout HeLa cells treated with siRNA against HIF-2α or that with negative control (Scr) under the indicated oxygen conditions for 24 h. Results are shown as mean ± s.d. (*n* = 4); ***P < 0.001, ****P < 0.0001 (Student’s *t* test). **I** Same experiments as in (**h**) were performed using HIF-2α knockout HeLa cells and siRNA against HIF-1α. Results are shown as mean ± s.d. (*n* = 4); ****P < 0.0001 (Student’s *t* test).

To identify the regulatory step for the hypoxic induction of HMHA1 expression, we first examined the importance of transcription initiation by inhibiting the binding of RNA polymerase to DNA with actinomycin D. The hypoxic induction of *VEGFA* and *CA9* was totally suppressed, meaning that transcription activity was attenuated by actinomycin D. Similarly, the induction of *HMHA1* mRNA expression under hypoxic condition was also completely abrogated upon actinomycin D treatment, suggesting that HMHA1 expression under hypoxia was indeed regulated at the transcription initiation step (Fig. 2a).

Since hypoxia-inducible factor (HIF) is one of the major transcription factors that govern hypoxia responses, we next examined whether HMHA1 expression is under the control of HIF. The degradation of HIF α-subunits under normoxic conditions is regulated by propyl-4-hydroxylase domain proteins (PHD), which belong to the O_2_/Fe(II)/2-oxoglutarate-dependent dioxygenase (2-OGDD) superfamily; we therefore treated the cells with deferoxamine (DFO), a Fe(II) ion chelator, or dimethyloxalylglycine (DMOG), a 2-oxoglutarate analogue, to inhibit 2-OGDD activities and prevent HIF α-subunit degradation. As expected, HIF-1α protein and the expression of the representative HIF-1 downstream gene, *CA9*, could be detected in the treated cells even under normoxia (Fig. 2b, c; Supplementary Fig. S2A, B), and HMHA1 expression was also induced in the treated cells (Fig. 2b, c).

In order to examine the dependence on HIF directly, we then performed loss-of-function studies. The CRISPR/Cas 9 system was exploited to establish clones of knockout (KO) HeLa cells using gRNAs that specifically target the *HIF1A*, *EPAS1*/*HIF2A*, or *ARNT* (HIF-1β) gene, respectively (Supplementary Fig. S3A–C). HIF-1β KO completely abrogated the induction of HMHA1 mRNA and protein expression under hypoxia (Fig. 2d). And, the reconstitution of HIF-1β into the KO cells rescued the induction of HMHA1 expression, suggesting that HIF is responsible for the hypoxic induction of HMHA1 (Fig. 2e). To identify the responsible HIF-α subunit, we first focused on HIF-1α, the major binding partner of HIF-1β. HIF-1α KO significantly suppressed HMHA1 induction under hypoxia, and such suppression was reversed by the reconstitution of HIF-1α (Fig. 2f, g). However, HIF-1α KO or knockdown (KD) did not completely abrogate HMHA1 expression upon hypoxic treatment; residual amount of HMHA1 was expressed under hypoxia, suggesting that the expression of HMHA1 is not solely regulated by HIF-1α (Fig. 2f, g; Supplementary Fig. S2C). We thus explored the involvement of another HIF-α isoform, HIF-2α, which may function in a compensatory fashion to reinstate HIF-1α-induced hypoxia responses. To test this hypothesis, we combined KO and KD against HIF-1α and HIF-2α. Whilst HIF-2α KD in parent HeLa cells only partially lowered HMHA1 induction, HIF-2α KD in combination with HIF-1α KO fully suppressed the hypoxic induction of HMHA1 (Fig. 2h; Supplementary Figure S2D). Similarly, HIF-1α KD in combination with HIF-2α KO obliterated the residual induction of HMHA1 observed in HIF-1α KO or KD alone (Fig. 2i; Supplementary Fig. S2E). Collectively, these data show that expression of HMHA1 is regulated at the mRNA level by both HIF-1 and HIF-2.

### HIF-1 is recruited to the intron 1 of *HMHA1* gene locus under hypoxia

**Fig. 3:**
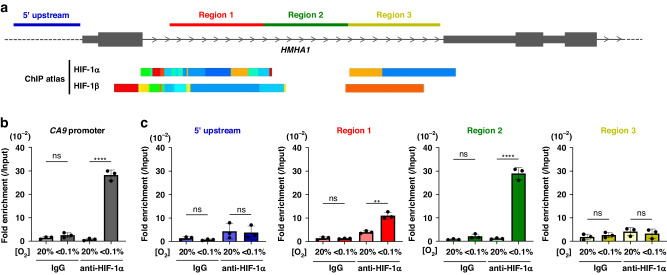
HIF-1 is recruited to the intron 1 of HMHA1 gene locus under hypoxia. **a** Schematic diagram showing the HMHA1 gene locus and the putative binding regions of HIF according to the ChIP Atlas (https://chip-atlas.org). qPCR primer sets were designed to flank the 5’ region upstream of Exon 1 (blue) and the three different regions of Intron 1 (Region 1–3: red, green, and yellow, respectively). **b** After HeLa cells were cultured under the indicated oxygen conditions for 24 h, chromatin immunoprecipitation (ChIP) was performed using anti-HIF-1α antibody or control IgG. The primer set for CA9 gene promoter region was used for qPCR. Results were normalised to the input samples. Results are shown as mean ± s.d. (*n* = 3); ns: not significant, ****P < 0.0001 (Student’s *t*-test). **c** Same experiments as (**b**) were performed using primer sets flanking the four regions depicted in (**a**) (coloured correspondingly) for ChIP-qPCR. Results are shown as mean ± s.d. (*n* = 3); ns: not significant, **P < 0.01, ****P < 0.0001 (Student’s *t* test).

Analyses with the ChIP Atlas database (https://chip-atlas.org) suggested that HIF-1α and HIF-1β can be recruited to the intron 1 of *HMHA1* gene locus (Fig. 3a). Thus, we next tested whether HIF-1α is actually recruited to the intronic region, and moreover, tried to narrow down the responsible region in it. HeLa cells were exposed to normoxic or severely hypoxic condition for 24 h, and then subjected to the ChIP-qPCR experiments (Fig. 3b, c) using anti-HIF-1α antibody for immunoprecipitation and primers flanking three regions of the *HMHA1* intron 1 for qPCR (Fig. 3a). After confirming the recruitment of HIF-1α to the *CA9* gene promoter region under the hypoxic condition (Fig. 3b), qPCR was performed using primer sets specific to the three different regions in the *HMHA1* locus (Region 1-3 in Fig. 3a). The amounts of genome DNA fragments containing Region 1 (136–269 bp, red) or Region 2 (251–508 bp, green) of intron 1 immunoprecipitated with anti-HIF-1α antibody were significantly increased under hypoxia, with the latter being particularly substantial (Fig. 3c). In contrast, there were no differences in the immunoprecipitated amounts of genome DNA fragments containing the 5’ upstream region (blue) of the *HMHA1* gene locus or the Region 3 (494–810 bp, yellow) of intron 1 between normoxia and hypoxia (Fig. 3c). These results together demonstrate the direct recruitment of HIF-1α to the *HMHA1* intron 1 locus, especially to the Region 2, possibly by which HIF regulates HMHA1 expression.

### HMHA1 is involved in the augmented cancer cell invasion under hypoxia by upregulating MMP-2 and MMP-9 activities

**Fig. 4:**
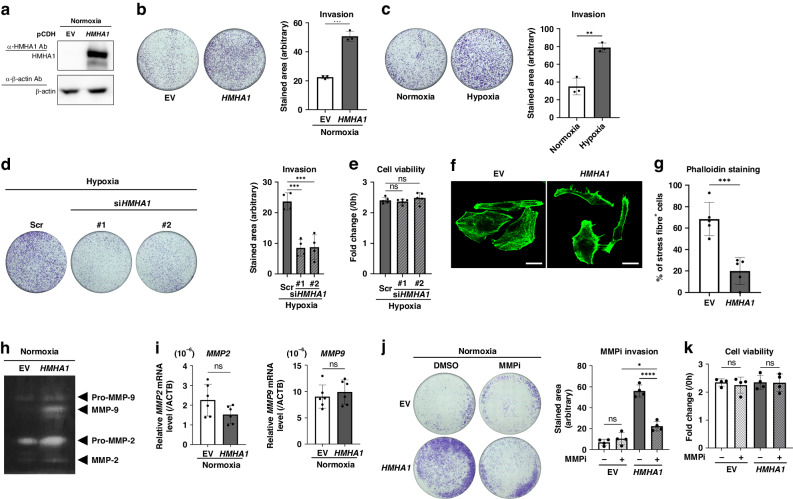
HMHA1 is involved in the augmented cancer cell invasion under hypoxia by upregulating MMP-2 and MMP-9 activities. **a**, **b** Stable transfectants of HeLa cells with HMHA1 overexpression expression vector or its corresponding empty vector (EV) were subjected to Western blotting for the indicated proteins and to invasion assay under normoxia (20% O_2_). Results are shown as mean ± s.d. (*n* = 3); ***P < 0.001 (Student’s *t* test). Representative images are shown. **c**, **d** HeLa cells (**c**) and HT1080 cells transfected with siRNA against HMHA1 (#1, #2) or its scramble control (Scr) (**d**) were used for the invasion assay under normoxia (20% O_2_) or hypoxia (1% O_2_). Results are shown as mean ± s.d. (*n* = 3 for **c**; *n* = 4 for **d**); **P < 0.01, ***P < 0.001 (Student’s *t* test). Representative images are shown. **e** Viability of HT1080 cells after HMHA1 knockdown treatment as in (**d**) was assessed. Results are shown as mean ± s.d. (*n* = 5); ns: not significant (Student’s *t* test). **f**, **g** HeLa cells overexpressing HMHA1 and the corresponding empty vector (EV)-transfected cells were stained with phalloidin (green). Scale bar: 20 µm. Reproducibility was confirmed in three independent set-ups and representative images are shown (**f**). Percentage of cells with prominent stress fibre structures (stress fibre^+^) in (**f**) was quantified (**g**). For each set-up, 5 random fields were studied; number of cells per field ranged between 7 and 21 cells, and at least 50 cells were examined. Results are shown as mean ± s.d. (*n* = 5); *** *P* < 0.001 (Student’s *t* test). **h** Conditioned media from HT1080 cells transiently transfected with HMHA1 overexpression vector or the corresponding empty vector (EV) were subjected to gelatin zymographic analyses. **i** qPCR (for *MMP2* and *MMP9* mRNA) using HeLa cells overexpressing HMHA1 and the corresponding empty vector (EV)-transfected cells. Results are shown as mean ± s.d. (*n* = 6); ns: not significant (Student’s *t* test). **j** HeLa cells overexpressing HMHA1 and the corresponding empty vector (EV)-transfected cells were subjected to invasion assay under normoxia (20% O_2_) in the presence of MMP-2/-9 inhibitor (MMPi, 2 µM) or DMSO control. Results are shown as mean ± s.d. (*n* = 4); * < 0.05, ****P < 0.0001, ns: not significant (Student’s *t* test). Representative images are shown. **k** Cell viability after MMP-2/-9 inhibitor (MMPi) treatment as in (**j**) was assessed. Results are shown as mean ± s.d. (*n* = 4); ns: not significant (Student’s *t* test).

After confirming the HIF-dependent HMHA1 induction, we investigated the effect of HMHA1 expression on the invasiveness of cancer cells under hypoxia by performing the Boyden chamber invasion assay. Compared to control cells transfected with the empty vector (EV), stable transfectant with HMHA1 overexpression vector showed an increased number of invading cells even under normoxia, illustrating that HMHA1 can indeed regulate the invasive properties of cancer cells (Fig. 4a, b). The stimulatory effect of hypoxia on invasion was then verified; results demonstrate that cancer cell invasion was significantly enhanced under hypoxic conditions (Fig. 4c). Moreover, in the loss-of-function experiment, siRNA-mediated knockdown of HMHA1 significantly reduced the number of invading cells under hypoxia compared to the scramble (Scr) control (Fig. 4d; Supplementary Fig. S4A). Of note, cell viability was not affected by the knockdown of HMHA1, indicating that the reduction of hypoxia-induced invasion by HMHA1 silencing was not due to cell death (Fig. 4e). Together, these data show that HMHA1 expression contributes to the augmented invasive properties of cancer cells under hypoxia.

Next, we sought to pursue the mechanism by which HMHA1 augmented cancer cell invasion. HMHA1 has previously been reported to function as a GTPase-activating protein (GAP) for RHOA and/or RAC1, both of which are key regulators of actin dynamics that govern cell motility [43, 44]. We therefore performed phalloidin staining to investigate how HMHA1 affects actin filament structures. In the EV control, cells with actin filaments organised into prominent stress fibre structures could be frequently observed (Fig. 4f, g). However, upon HMHA1 overexpression, the actin filaments appeared to be more distorted and there were significantly less cells having intact stress fibre networks (Fig. 4f, g). These results are in line with previous reports, supporting that HMHA1 can regulate actin dynamics and affect cytoskeleton structures.

We then examined one of the important facets of cancer cell invasion, extracellular matrix (ECM) degradation. Since a major type of proteases responsible for such process is the matrix metalloproteinases (MMPs), in order to investigate whether HMHA1 can alter MMP activities, we performed a gelatin zymography assay. With the same amount of total extracellular proteins, the conditioned medium from HMHA1-overexpressing cells showed much higher gelatinolytic activities by MMP-2 and MMP-9 when compared to that from EV-transfected control cells, despite that their mRNA levels remained unchanged (Fig. 4h, i; Supplementary Fig. S4B). These results suggest that HMHA1 may promote invasion by regulating extracellular MMP-2/-9 activities. To test this hypothesis, we inhibited MMP-2/-9 activities with specific inhibitor and assessed the invasion of HMHA1-overexpressing cells. Whilst the overexpression of HMHA1 increased the number of invading cells, MMP-2/-9 inhibitor (MMPi), without compromising cell survival, largely reversed such increase, indicating that MMP-2/-9 activity is required for HMHA1 to enhance invasion (Fig. 4j, k). Taken together, these data show that HMHA1 augments cancer cell invasion in an MMP-2/-9-dependent manner.

### Ionising radiation under hypoxia followed by reoxygenation further enhances HMHA1 expression *via* the ROS/HIF axis and promotes cancer cell invasion

**Fig. 5:**
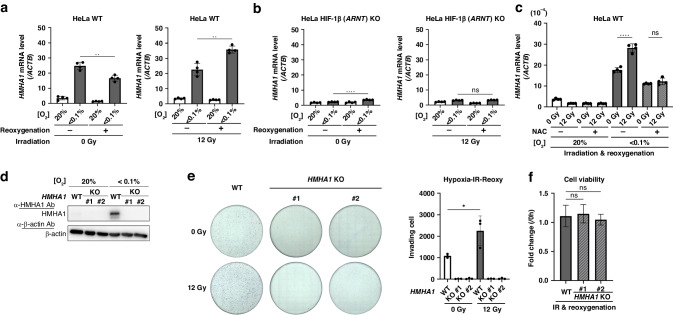
Ionising radiation under hypoxia followed by reoxygenation further enhances HMHA1 expression *via* the ROS/HIF axis and promotes cancer cell invasion. **a** HeLa cells incubated under the indicated oxygen conditions for 24 h were irradiated with the indicated doses of γ-ray, reoxygenated, further cultured for 6 h, and subjected to qPCR analysis for *HMHA1* mRNA. Results are shown as mean ± s.d. (*n* = 4); **P < 0.01 (Student’s *t* test). **b**, **c** Same experiments as (**a**) were performed using HIF-1β knockout HeLa cells (**b**), or parent HeLa cells (WT) in the presence or absence of NAC (5 μM) (**c**). Results are shown as mean ± s.d. (*n* = 4); ns: not significant, ****P < 0.0001 (Student’s *t* test). **d** Parent HeLa cells (WT) and two clones of HMHA1 knockout HeLa cells (KO#1 and #2) were cultured under the indicated oxygen conditions for 24 h and subjected to Western blotting for the indicated proteins. **e** Parent HeLa cells (WT) and HMHA1 knockout clones (KO#1 and #2) were irradiated under hypoxic conditions (< 0.1% O_2_) with the indicated doses of γ-ray, reoxygenated, and subjected to the Boyden chamber invasion assay. Results are shown as mean ± s.d. (*n* = 3); *P < 0.05 (Student’s *t* test). **f** Cell viability after irradiation-reoxygenation treatment as in (**e**) was assessed. Results are shown as mean ± s.d. (*n* = 6); ns: not significant (Student’s *t* test).

We previously reported that after severely hypoxic tumour cells in distal regions of blood vessels in tumour tissues survive radiation therapy, they acquire HIF-1 activity due to reoxygenation and the resulting increase in reactive oxygen species (ROS) amount, and subsequently translocate to proximal regions of tumour blood vessels [37, 45]. Therefore, we next examined whether HMHA1 expression is also increased after irradiation-reoxygenation treatment that closely mimics the in vivo condition. Strikingly, whilst irradiation alone did not induce HMHA1 expression, irradiation under hypoxia and postirradiation reoxygenation conjointly elicited a further induction of HMHA1 expression (Fig. 5a). Moreover, upon HIF-1β knockout, there were no changes in HMHA1 levels even after the same irradiation-reoxygenation treatment, indicating that irradiation followed by reoxygenation treatment further induces HMHA1 expression in a HIF-dependent manner (Fig. 5b).

Considering that irradiation and reoxygenation can both stimulate reactive oxygen species (ROS) generation, we hypothesised that the increase in HMHA1 expression by the irradiation and reoxygenation treatment might have been resulted from ROS-mediated stabilisation of the HIF-1α protein. To verify this, HeLa cells were pre-treated with a ROS inhibitor, NAC (N-acetyl-L-cysteine). Without the NAC treatment, irradiated cells showed higher HMHA1 expression than non-irradiated cells after reoxygenation (Fig. 5c). On the other hand, in the NAC-treated group, the irradiation-reoxygenation treatment had no effects on HMHA1 expression levels, demonstrating that ROS is indeed involved in the induction of HMHA1 expression after irradiation and reoxygenation. Collectively, these results delineated the induction of HMHA1 in irradiated ex-hypoxic cells *via* the ROS/HIF axis.

Last, to examine whether induction of HMHA1 expression by irradiation-reoxygenation treatment can effectuate changes in invasive properties of cancer cells, we employed the Boyden chamber invasion assay again using the parent HeLa cells (WT) and HMHA1 KO HeLa cells (Fig. 5d; Supplementary Fig. S5). The cells were irradiated under hypoxic conditions (<0.1% O_2_) followed by reoxygenation treatment, and then subjected to the assay. As expected, for WT cells, there were more invading cells in the “irradiated reoxygenated group” than the “non-irradiated reoxygenated control group” (Fig. 5e). On the other hand, in HMHA1 KO cells, which only exhibited modest invasion activity under hypoxic conditions even without irradiation treatment, the stimulatory effect of irradiation-reoxygenation on invasion was not observed, revealing that HMHA1 is required for cancer cell invasion after irradiation and reoxygenation (Fig. 5e). Moreover, the WT cells and HMHA1 KO clones did not differ in their viability after irradiation and reoxygenation treatment, indicating that the decrease in invasion was not due to cell death or differences in radiosensitivity (Fig. 5f). Together, our results collectively provided a plausible molecular mechanism underlying the invasion of hypoxic cancer cell elicited by irradiation-reoxygenation in a ROS-HIF-HMHA1-dependent manner.

### Tumour hypoxia signature is associated with both HMHA1 expression levels and poor overall survival in various types of cancer patients

**Fig. 6:**
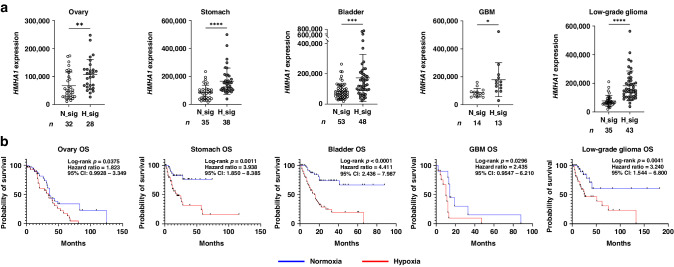
Tumour hypoxia signature is associated with both HMHA1 expression levels and poor overall survival in various types of cancer patients. **a** *HMHA1* expression levels in normoxic (with low hypoxia-signature) and hypoxic (with high hypoxia-signature) tumours. Results are shown as mean ± s.d. (The numbers of sample are indicated below each graph.); *P < 0.05, **P < 0.01, ***P < 0.001, ****P < 0.0001 (Student’s *t* test). **b** TCGA-based Kaplan-Meier analysis of overall survival of patients with ovarian cancer, stomach cancer, bladder cancer, glioblastoma (GBM), and low-grade glioma stratified by the intratumoral oxygen environments (normoxia: low hypoxia signature; hypoxia: high hypoxia signature). The censored cases are shown as ticks on the line. *P*-values, hazard ratios, and confidence intervals (95% CI) are indicated in each graph (Log-rank test).

Finally, we sought to extrapolate from our findings the clinical significance of the hypoxic induction of HMHA1. Using the gene expression data from The Cancer Genome Atlas (TCGA), patients were first segregated by their expression of the hypoxia signature genes [46]. In various cancer types, we found that patient samples with high expression of hypoxia signature genes, which implies tumour hypoxia, had significantly higher expression levels of HMHA1, when compared to those with low hypoxia signature expression (Fig. 6a). Furthermore, Kaplan–Meier analysis revealed that such high expression levels were associated with poor overall survival of various types of cancer patients (Fig. 6b). Taken together, these data support that HMHA1 expression is induced by hypoxia and is associated with worse clinical outcomes in cancer patients.

## Discussion

Hypoxia has been reported to promote malignant properties including angiogenesis, metabolic reprogramming, cancer stem cell phenotypes, immune evasion, invasion and metastasis by increasing the expression of HIF-regulated genes [25]. As a result, tumour hypoxia contributes to therapy resistance and is associated with poor prognosis in patients. Whilst radiotherapy remains one of the most effective modalities for cancer treatment, biological and clinical studies have also revealed that significant proportion of patients still developed distant metastasis after receiving radiotherapy [47]. In addition, radiation treatment has been reported to enhance cancer cell invasion, as well as be associated with incidence of distant metastasis in some cancer patients [34, 48–50]. In this study, we have identified a novel HIF downstream gene, *HMHA1*, whose expression was induced under hypoxia and thereby augmented the invasion activity of hypoxic cancer cells. Moreover, we found that irradiation followed by the reoxygenation of hypoxic cancer cells led to a further increase in HMHA1 expression *via* the ROS/HIF pathway, subsequently enhancing the invasion of surviving ex-hypoxic cells.

Our results first demonstrated that HIF-1β knockout completely abrogated the hypoxia-induced expression of HMHA1, which could be recapitulated by the simultaneous suppression of HIF-1α and HIF-2α, but not the knockout of HIF-1α or HIF-2α alone. This suggests that there potentially exists a compensatory mechanism between HIF-1 and HIF-2, which functions to secure HIF-mediated hypoxia responses even either of the α subunits is not available; however, the molecular details of such compensation as well as the selectivity of HIF-1α and HIF-2α to gene promoters are still elusive. Moreover, whilst our ChIP-qPCR data showed the recruitment of HIF-1 to the HMHA1 intronic region, the exact hypoxia response element remains to be elucidated.

The HMHA1 protein mainly comprises a F-BAR domain, a C1 domain, and a GTPase-activating protein (GAP) domain, and is normally highly expressed in blood cells and immune cells. Previously, it has been reported that whereas the forced expression of an artificially truncated form of HMHA1, which does not contain the N-terminal F-BAR domain, could regulate GTPase activity and alter cell morphology (cell spreading), the full-length HMHA1 did not exert any effects in HeLa cells, nor did the knockdown of HMHA1 affect the chemotactic migration activities of Jurkat cells [43]. On the other hand, a more recent study using knockout mice demonstrated that HMHA1 loss would compromise the deformability and trans-endothelial migration of naïve T cells [44]. Herein, we showed that the stable overexpression of full-length HMHA1 is functional and sufficient to enhance the invasion of cancer cells even under normoxic conditions. Such inconsistency may be attributed to the differences in cellular context and environmental cues, suggesting that HMHA1 regulates cell movement/translocation in response to particular stimuli or under specific conditions, rather than being generally involved in all migratory/cellular movement activities.

Whilst de Kreuk et al. depicted a model that the F-BAR domain acted in an auto-inhibitory manner to suppress RhoGAP activity, He et al. postulated an alternative model that the F-BAR and C1 domains sequester HMHA1 to the cell membrane and RhoGAP functions independently to regulate actin dynamics [43, 44]. In our IHC analysis, HMHA1 indeed appeared to localise to the cell membrane to certain extent (Fig. 1e); however, whether HMHA1 expressed under hypoxic conditions acts through one of the aforementioned models or not remains to be examined.

In this study, we sought to investigate the molecular events occurring after the hypoxic induction of HMHA1 that augmented invasion, but the details were not fully clarified. We showed that the overexpression of HMHA1 resulted in the distortion of stress fibre organisation; this is in line with He et al.’s previous findings which demonstrated that HMHA1 is required for cell deformability [44], and may potentially act as a mechanism for hypoxic cancer cells to enhance invasiveness. Moreover, HMHA1 overexpression remarkably enhanced the total gelatinolytic activities by extracellular MMP-2 and MMP-9, suggesting that HMHA1 may promote ECM degradation and thereby facilitates hypoxic cancer cell invasion. However, the increase in MMP-2 and -9 activities was not grounded in any changes in their mRNA expression levels, indicating that HMHA1 regulates MMP-2 and -9 posttranscriptionally. Online interactome database (http://www.interactome-atlas.org/) has reported the interaction between HMHA1 and proteins related to vesicular transport from Golgi, like RINT1, GOLGA2 and BICD2, raising the possibility that HMHA1 may be involved in the regulation of MMP-2 and -9 secretion, but further efforts to elucidate the regulatory mechanism would be necessary. In addition, it may also be worthy to explore whether the roles of HMHA1 in regulating actin fibre structures and in regulating MMP activities are related to each other and if they are functioning synergistically to promote metastasis.

Of note, our data showed that cancer cell invasion under hypoxia was drastically, but not completely, suppressed by the knockdown of HMHA1. This result seems reasonable based on previous findings that HIF can enhance invasiveness via other independent pathways like the HGF/MET [51, 52] and the LOX/LOXL2/LOXL4 pathways [40].

Severely hypoxic cancer cells located in perinecrotic regions of tumour tissues acquire HIF-1 activity after surviving radiation therapy in a reoxygenation- and the resulting ROS-dependent manner [28, 45]. We previously reported that the postirradiation HIF-1 activation is responsible for invasion of surviving cells towards tumour blood vessels, tumour recurrence, and potentially distant tumour metastases after radiation therapy [37]; however, its key regulator remained elusive. In the present study, we found that HMHA1 expression is induced after irradiation-reoxygenation treatment in a ROS/HIF-dependent manner, and HMHA1 is involved in promoting the invasion of irradiated and subsequently reoxygenated ex-hypoxic cancer cells. Our results provide an insight into the molecular basis behind post-radiotherapy invasion, distant metastasis, and tumour recurrence.

Last, herein, we exploited the Boyden chamber transwell assay to evaluate the invasiveness of cancer cells. Whilst this is one of the standard in vitro assays to study cell invasion activity, as introduced before, metastasis is a cascade of biological events. Therefore, it is important to establish suitable HMHA1 KO cell lines and perform in vivo studies in order to assess whether HMHA1 can indeed enhance metastasis. In addition, it is also worthy to subject HMHA1 KO xenograft tumours to irradiation treatment and investigate whether abrogating HMHA1 can halt the HIF-dependent translocation of severely hypoxic cancer cells towards tumour blood vessels, and prevent tumour recurrence and distant metastases after irradiation.

## Materials and methods

### Cell culture and reagents

All cell lines used were purchased from American Type Culture Collection (ATCC). HeLa, HepG2, SBC-3, and HT1080 cells were cultured in Dulbecco’s Modified Eagle Medium (DMEM) with high glucose (4.5 g/L-Glucose), L-Gln, and sodium pyruvate (Nacalai Tesque, Cat. #08458-16) supplemented with 10% (v/v) FBS (EQUITECH-BIO, Cat. #268-1) and 1% (v/v) penicillin-streptomycin. Cells were maintained at 37 ˚C in a 95% air/5% CO_2_ moisturised incubator. For hypoxic treatment, cells were cultured at 37 ˚C in workstation chambers maintained at < 0.1% O_2_/5% CO_2_ (INVIVO_2_ 500, Ruskinn), at 1% O_2_/5% CO_2_, or at 3% O_2_/5% CO_2_ (INVIVO_2_ 400, Ruskinn). We prepared both “< 0.1% O_2_” and “1–3% O_2_” conditions in the present study since they have been recognized as “severely hypoxic conditions” and “relatively mild hypoxic conditions”, respectively, in the fields of radiation oncology and radiation biology. Gene silencing was performed using Lipofectamine™ RNAiMAX Transfection Reagent (Invitrogen, Cat. #13778075) and siRNAs purchased from Life Technologies; siRNAs against HIF-1α (HSS104775 and HSS179231), HIF-2α (HSS176568 and HSS176569), and HMHA1 (HSS118888 and HSS118890) were used. Stealth RNAi™ siRNA Negative Control, Med GC (Invitrogen, Cat. #12935300) was used as negative control. A MMP inhibitor (MMPi), MMP-2/MMP-9 Inhibitor I, was purchased from Cayman Chemical (Cat. #20315).

### Plasmid construction and stable transfectant

To construct HMHA1 overexpression vector, reverse transcription of total RNA from HeLa cells was performed using the RNA LA PCR™ Kit (AMV) Ver.1.1 kit (Nacalai tesque, Cat. #RR012A) in accordance with manufacturer’s protocol. HMHA1 coding sequence was amplified from the resultant cDNA using the following primers: 5’-ATAGGATCCGCCATGAGTCGGGGGCAAAG-3’ and 5’-ATAGCGGCCGCTCACACGAATTCCGGCTG-3’, and inserted between the BamHI and NotI sites of the pCDH/EF1.MCS-IRES.Puro vector (System Biosciences, Cat. #CD532A-2). HEK293TN cells were co-transfected with pCDH/EF1α.MCS-IRES.Puro empty vector or pCDH/EF1α.HMHA1-IRES.Puro (for HMHA1 overexpression) in combination with pVSV-G, pPACKH1/GAG and pPACKH1/REV, and then cultured for 2 days. Lentivirus-containing medium was filtered through 0.22 μm pore size membrane (Millipore, Cat. #SLGSV255F) and applied to sparsely seeded HeLa cells pre-incubated with polybrene (8 μg/mL). After 2 days, cells were subjected to puromycin selection for 2–3 weeks to obtain bulks of transduced cells.

### CRISPR/Cas9-mediated establishment of knockout cells

HIF-1α and HIF-2α knockout HeLa cells were generated using sgRNA oligonucleotides targeting the upstream of exon 1 and inside intron 1 (HIF-1α KO clone #1: 5’-TCGCTCGCCATTGGATCTCG-3’ and 5’-TCTAATGGTGTCACGGCTCA-3’, respectively; HIF-1α KO clone #2: 5’-TGATTGGCTGAGAGCGGCGT-3’ and 5’-GGTGCTCTGCAGTGGACCGT-3’, respectively; HIF-2α KO: 5’-ACAGGCAACGGTTAGCGCTC-3’ and 5’-AGCGGGCGTCCGGGCCGATC-3’, respectively) as per the described procedures [53]. Previously established HIF-1β knockout HeLa cells were used [53]. HMHA1 knockout cells were generated using sgRNA oligonucleotides targeting the coding sequence of HMHA1 (sgRNA #1: 5’-GCGTCTGCGAGATCGAGCGG-3’; #2: 5’-AGTGGACCGTTCCGCCACGA-3’). The oligonucleotides were inserted into the lentiCRISPRv2-puro vector (Addgene, Cat. #98290) for co-transfection together with the packaging plasmids pVSV-G, pPACKH1/GAG, and pPACKH1/REV. Transduced cells were selected as aforementioned and sparsely reseeded for isolation of single clones. KO clones were verified by Western blot analysis and sequencing of the corresponding *HMHA1* gene loci (Supplementary Fig. S5).

### Microarray analysis

The GeneChip system with a Human Genome U133-plus 2.0 array spotted with 54,675 probe sets (Affymetrix Inc.) was used for the microarray gene expression analysis, according to the manufacturer’s instructions. Briefly, cRNA was synthesised from 500 ng of total RNA using the GeneChip 3’ IVT Express Kit (Affymetrix Inc.). Fragmented cRNA labelled with biotin was hybridized to the array at 45 °C for 16 h. After stained with streptavidin-phycoerythrin, the array was scanned with a probe array scanner, and the significant genes of interests were extracted from the obtained data about hybridisation intensity by using the GeneSpring GX software (Agilent Technologies Inc.). The microarray dataset was deposited in the NCBI’s Gene Expression Omnibus database (GEO) with the accession number GSE161393 [38].

### RNA-Seq analysis

After cells were cultured under normoxic or hypoxic conditions for 24 h, total RNA was harvested and purified from the cells using TRIZOL reagent (Ambion, Cat. #15596026). The sample quality was analysed with Bioanalyzer (Agilent Technologies) and TapeStation (Agilent Technologies), and the samples were subjected to directional library synthesis (NEB, Cat. #E7420) and subsequently to the library quantification using the bioanalyzer DNA High-sensitivity kit (Agilent, Cat. #5067-4626). RNA sequencing was then conducted using Illumina NextSeq500 (Illumina) in Tsukuba i-Laboratory at Tsukuba University. Acquired dataset was deposited in the Gene Expression Omnibus (GEO) database at the National Center for Biotechnology Information (NCBI) (GEO accession number: GSE254480).

### Reverse transcription and quantitative PCR (qPCR)

Cells were lysed in Sepasol-RNA I Super G (Nacalai tesque, Cat. #09379-84) and 1 µg of the extracted RNA was reverse-transcribed using the PrimeScript RT Reagent Kit (Takara, Cat. #RR037A) in accordance with manufacturer’s instructions. Real-time qPCR was performed using the TB Green Premix Ex Taq II and analysed with the Thermal Cycler Dice Real Time System (Takara). The mRNA expression levels of target genes relative to β-actin (*ACTB*) were assessed. The primer sequences are as listed in Supplementary Table S4.

### Chromatin immunoprecipitation (ChIP)-qPCR

1.5 × 10^6^ cells (per 10 cm dish) were cultured under normoxia or hypoxia for 24 h, and formalin solution was added to create DNA-protein cross-link. After quenching with glycine (136 mM), cells were washed twice with ice-cold PBS and harvested with 300 µL SDS Lysis Buffer (Merck, Cat. #20-163). Cell lysates were sonicated on ice and then centrifuged at 15,000 × g to remove the cell debris. Supernatant was diluted with ChIP Buffer (Merck, Cat. #20-153) and immunoprecipitation was performed with anti-HIF-1α antibody (Abcam, Cat. #ab1) or mouse IgG1 κ isotype control (BD Pharmingen, Cat. #554121) using the Dynabeads Protein G Immunoprecipitation Kit (Thermo Fisher, Cat. #10007D). After overnight incubation on a rotating platform at 4 ˚C, magnetic beads were sequentially washed with Low Salt Buffer, High Salt Buffer, LiCl Buffer and TE buffer (Merck, Cat. #20-154, 20-155, 20-156; Nacalai tesque, Cat. #06890-25). DNA was eluted in 1% (w/v) SDS with 100 mM NaHCO_3_, and cross-links were reversed by adding NaCl (final concentration 200 mM). DNA was purified with the QIAquick PCR purification kit (QIAGEN, Cat. #28106) before qPCR analysis of candidate HIF binding regions. Primers used for qPCR are listed in Supplementary Table S4.

### Western blotting

Whole-cell lysates were prepared with Cell Lytic M (Sigma-Aldrich, Cat. #C2978) and equal amounts of protein extract were loaded to each well of the SDS-containing acrylamide/bisacrylamide gel for SDS-PAGE. After electroblotting proteins from the gel onto PVDF blotting membrane (0.45 µm pore size, Amersham Cytiva, Cat. #10600023), the membrane was blocked with 5% (w/v) skim milk in TBS-T and target proteins were probed with the corresponding primary antibodies, anti-HMHA1/ARHGAP45 rabbit polyclonal antibody (Atlas Antibodies, Cat. #HPA019816, 1:300 dilution), anti-HIF-1αmouse monoclonal antibody (BD Biosciences, Cat. #610959, 1:250 dilution), anti-EPAS1/HIF-2α mouse monoclonal antibody (Santa Cruz, Cat. #sc-13596, 1:200 dilution), anti-HIF-1β mouse monoclonal antibody (Novus Biologicals, Cat. #NB100-124, 1:1000 dilution), and anti-β-actin mouse monoclonal antibody (Santa Cruz, Cat. #sc-69879, 1:200 dilution), at 4 ˚C overnight and subsequently with HRP-conjugated secondary bodies, anti-mouse IgG whole antibody (Cytiva, Cat. #NA931), and anti-rabbit IgG whole antibody (Cytiva, Cat. #NA934), at room temperature for 2 h. Chemiluminescence signals were developed by adding ECL reagents (Amersham Cytiva, Cat. #RPN2232) and visualised with the Amersham™ Imager 680 system (Amersham Cytiva).

### In vivo experiments

All animal experiments were approved by the Animal Research Committee of Kyoto University. We performed all experiments according to the guidelines governing animal care in Japan. Eight-week-old female athymic nude mice (BALB/c *nu*/*nu*) were purchased from SLC Inc. Wild-type HeLa cells or HMHA1 knockout HeLa cells were subcutaneously transplanted into the right hind leg (1 × 10^6^ cells in 100 µL ice-cold PBS per mouse). To reduce oxygen supply to the xenografted tumour, phenylhydrazine hydrochloride (60 mg/kg body weight) was injected intraperitoneally thrice with 1-day intervals before the mice were euthanised by cervical dislocation. After the entire tumour and the left kidney were surgically excised and homogenised in Sepasol RNA I Super G (Nacalai tesque, Cat. #09379-84) using the TissueLyser LT (QIAGEN), total RNA was extracted for qPCR analyses. Alternatively, the tumour-bearing mice were injected with pimonidazole hydrochloride (Hypoxyprobe, Inc., Cat. #HP1), a hypoxia marker, at a dose of 60 mg/kg body weight 1 h before euthanasia to allow for staining of hypoxic regions in tumour tissues in accordance with the manufacturer’s instructions.

### Immunohistochemical (IHC) analyses

Serial sections were prepared from formalin-fixed paraffin-embedded (FFPE) tumour tissue and subjected to IHC staining using FITC-conjugated anti-pimonidazole mouse monoclonal antibody (Hypoxyprobe, Inc., Cat. #HP2-100, 1:200 dilution) and anti-HMHA1 rabbit polyclonal antibody (Atlas Antibodies, Cat. #HPA019816, 1:100 dilution) as primary antibodies, and Alexa Flour 594 goat anti-rabbit IgG (Invitrogen, Cat. #A-11072, 1:1000 dilution) as secondary antibody. Procedures were as per previously described [54]. Reproducibility was confirmed using sections of xenografted tumours from three individual mice and representative images were shown.

### Phalloidin staining

Cells were seeded to collagen-coated glass bottom dishes (Matsunami Glass, Cat. #D11134H) and incubated for 24 h before fixation with 4% paraformaldehyde solution. Fixed cells were stained with green fluorescein-conjugated phalloidin (AAT Bioquest, Cat. #23115) in accordance with manufacturer’s protocol. Fluorescence images were obtained with the FLUOVIEW FV10i confocal laser scanning microscope (Olympus Life Science). At least 5 fields were randomly imaged for each sample, and the presence of organised stress fibre was evaluated by an independent blinded assessor.

### Irradiation

Cells were cultured under normoxic or hypoxic (O_2_ < 0.1%) conditions for 24 h before irradiation with the indicated dose of ^137^Cs γ-rays using the Gammacell 40 Exactor (MDS Nordion International Inc.). N-acetyl-L-cysteine (NAC) was added at the final concentration 5 mM (Sigma-Aldrich, Cat. #A9165) 1 h prior to irradiation. Hypoxia was maintained during irradiation by transferring cells from the hypoxia workstation chambers to a rectangular jar of AnaeroPack (Mitsubishi Gas Chemical) and cells were reoxygenated afterwards.

### Colorimetric cell viability assay

Cells transfected with the scramble control or siRNA against HMHA1 were seeded to a 96-well plate (8 × 10^3^ cells/well) and cultured for 24 h. Cell viability was determined using the Cell Count Reagent SF kit (Nacalai Tesque, Cat. #07553-44) in accordance with the manufacturer’s protocol.

### Boyden chamber invasion assay

Cells were pre-treated with serum-reduced DMEM medium (1% FBS, for HT1080) or serum-free DMEM medium (0% FBS, for HeLa) overnight before seeding into the upper well (2 × 10^4^ cells/well) of the Matrigel-coated transwell insert (BioCoat Matrigel Invasion Chambers, 24-well Plates, Corning, Cat. #354480). The starved cells were allowed to adhere for 12 h before changing the medium in the bottom well from serum-free DMEM to serum-supplemented (10% FBS) DMEM. Afterwards, cells were incubated in the indicated conditions for 24 h, and fixed with 4% paraformaldehyde. Non-invading cells remained in the upper chamber were removed by wiping with clean cotton buds. After staining with crystal violet solution (0.5% w/v in 20% methanol), images of the entire membrane were captured with the Keyence BZ-9000 system and invading cells were quantified by ImageJ analysis.

### Gelatin zymography

To assess the enzymatic activities of excreted MMP-2/-9, HT1080 cells transfected with empty vector or HMHA1 overexpression vector and cultured with DMEM (5% FBS) for 48 h under normoxia. The culture media were centrifuged and electrophoresed in 7.5% acrylamide/bisacrylamide gel containing 1.25 mg/mL gelatin. Loading amount was normalised by total protein amount of cell lysate. SDS was removed by washing the gel with 2.5% Triton X-100 in Tris-HCl buffer (pH 8.0) before incubation at 37 ˚C in Tris-HCl (pH 8.0) buffer containing 0.5 mM CaCl_2_ and 1.0 μM ZnCl_2_ overnight. Coomassie Blue staining was then performed to visualise gelatinolytic activity as transparent bands. For loading control, samples were applied for SDS-PAGE without gelatin, and the gel was stained with Coomassie brilliant blue to visualise protein bands.

### TCGA analysis

An open-access dataset of TCGA was obtained from GDC Data Portal (https://portal.gdc.cancer.gov). Using the 24 hypoxia-inducible genes, which were reported to be useful to select cancer patients with tumours exhibiting high hypoxia-signature by Yang et al. [46], cancer patients exhibiting high and low hypoxia-signature were selected from the TCGA dataset as follows. The patients with expression levels of 6 or more of the 24 hypoxia-inducible genes in the bottom 10% in the cohort were selected as the low hypoxia-signature (normoxic tumours) group, whereas those with expression levels of 6 or more of the 24 genes in the top 10% in the cohort were selected as the high hypoxia-signature (hypoxic tumours) group. Overall survival of ovarian cancer, stomach cancer, bladder cancer, glioblastoma (GBM), and low-grade glioma patients in each group was estimated by the Kaplan-Meier method and compared by log-rank test. Hazard ratio was computed with the GraphPad Prism software.

### Statistical analysis

Statistical analyses were conducted using GraphPad Prism software. Results are shown as the mean ± s.d. The significance of differences was assessed using the Student’s *t*-test and Log-rank test (ns: not significant, *P < 0.05, **P < 0.01, ***P < 0.001, ****P < 0.0001). The variance was confirmed to be similar between the groups that were being statistically compared. Appropriate sample sizes in each in vitro and in vivo experiment were determined based on previous experiments conducted in our laboratory, preliminary experiments conducted prior to this research, and previous literatures on the subject. Reproducibility of the results was confirmed in at least three independent experiments and representative results are shown.

### Supplementary information

Supplementary Figure S1-S5

Supplementary Table S1

Supplementary Table S2

Supplementary Table S3

Supplementary Table S4

## Data Availability

The authors declare that the data supporting the findings of this study are available within the paper and its supplemental information files. The data sets of microarray and RNA-Seq analyses were deposited in the NCBI’s Gene Expression Omnibus database with the accession number GSE161393 and GSE254480, respectively.

## Materials availability

Materials used in this study are available from the corresponding author upon reasonable request.
