# Supplementary material for: Hypoxia- and Postirradiation reoxygenation-induced HMHA1/ARHGAP45 expression contributes to cancer cell invasion in a HIF-dependent manner
Source: Br J Cancer. 2024 May 13;131(1):37–48. doi: 10.1038/s41416-024-02691-x (PMC11231347; doi:10.1038/s41416-024-02691-x)
Supplement: Supplementary file 1 — Supplementary Figure S1-S5 [file 41416_2024_2691_MOESM1_ESM.pdf]

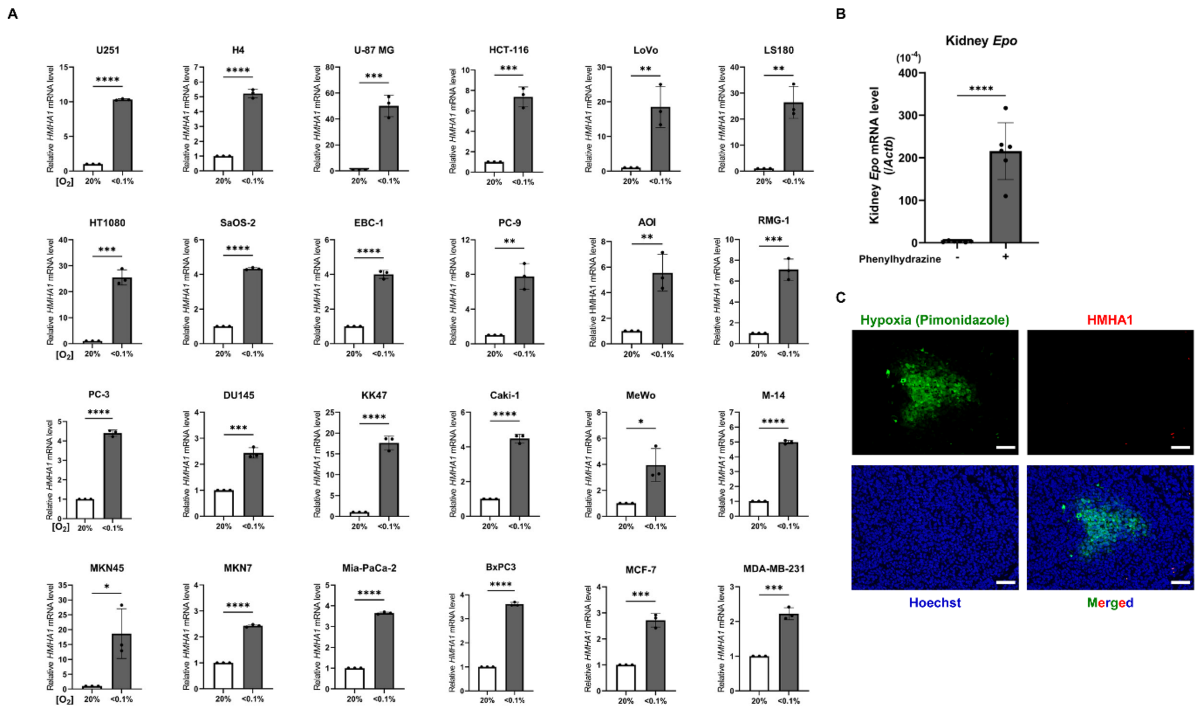

Supplementary Figure S1

A. qPCR for *HMHA1* mRNA using the indicated cancer cell lines after treatment at the indicated oxygen concentration. Results are shown as mean ± s.d. (n = 3); * $P < 0.05$, ** $P < 0.01$, *** $P < 0.001$, **** $P < 0.0001$ (Student's *t* test).

B. qPCR for *Epo* mRNA using kidneys of untreated or phenylhydrazine-treated mice. Results are shown as mean ± s.d. (n = 6); **** $P < 0.0001$ (Student's *t* test).

C. FFPE sections of HMHA1-KO HeLa tumour xenografts were double-stained with antibodies against the hypoxia marker, pimonidazole (green), and against HMHA1 (red). Nucleus was counterstained with Hoechst 33342 (blue). Scale bar: 50 µm. Reproducibility was confirmed in xenografted tumours from three independent mice and representative images are shown.

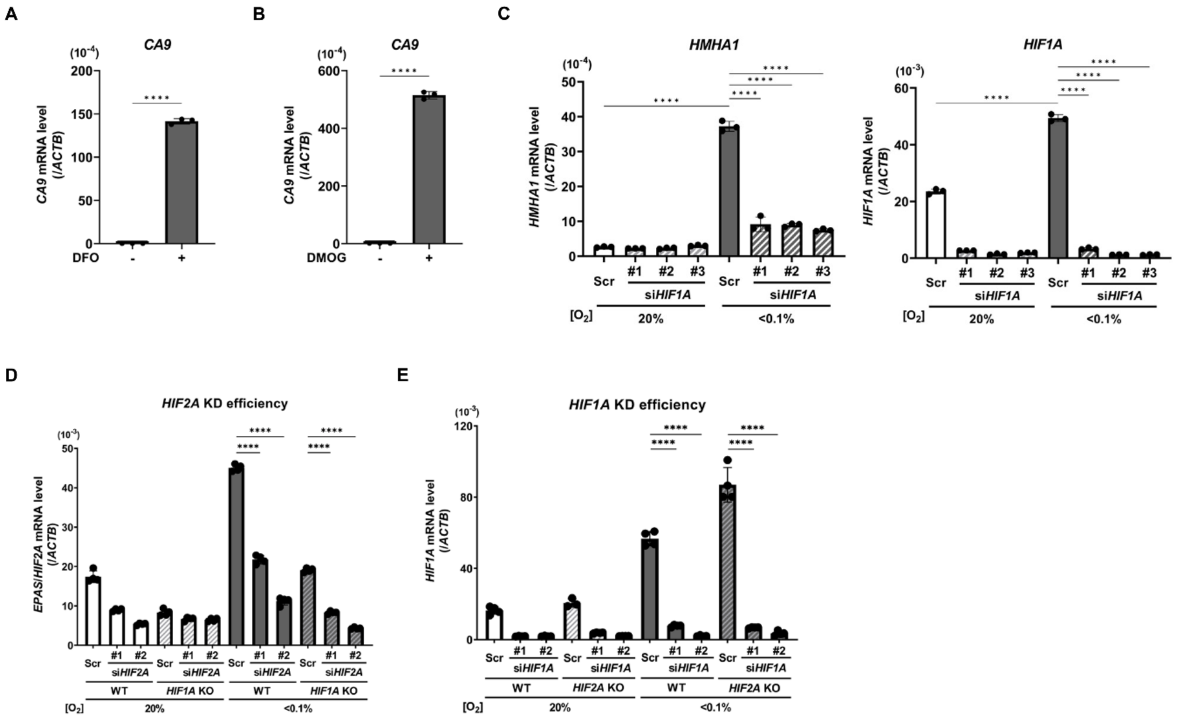

Supplementary Figure S2

A,B. qPCR for *CA9* mRNA using HeLa cells with (+) or without (-) of DFO (**A**) or DMOG (**B**) treatment. Results are shown as mean ± s.d. (n = 3); **** $P < 0.0001$ (Student's *t* test).

C. siRNA-mediated knockdown of HIF1A was performed for HeLa cells before incubation under the indicated oxygen condition for 24 hours. *HMHA1* mRNA levels were determined by qPCR. Results are shown as mean ± s.d. (n = 3); **** $P < 0.0001$ (Student's *t* test).

D,E. Efficiencies of siRNA-mediated knockdown of HIF2A (**D**) or HIF1A (**E**) were verified by qPCR. Results are shown as mean ± s.d. (n = 4); **** $P < 0.0001$ (Student's *t* test).

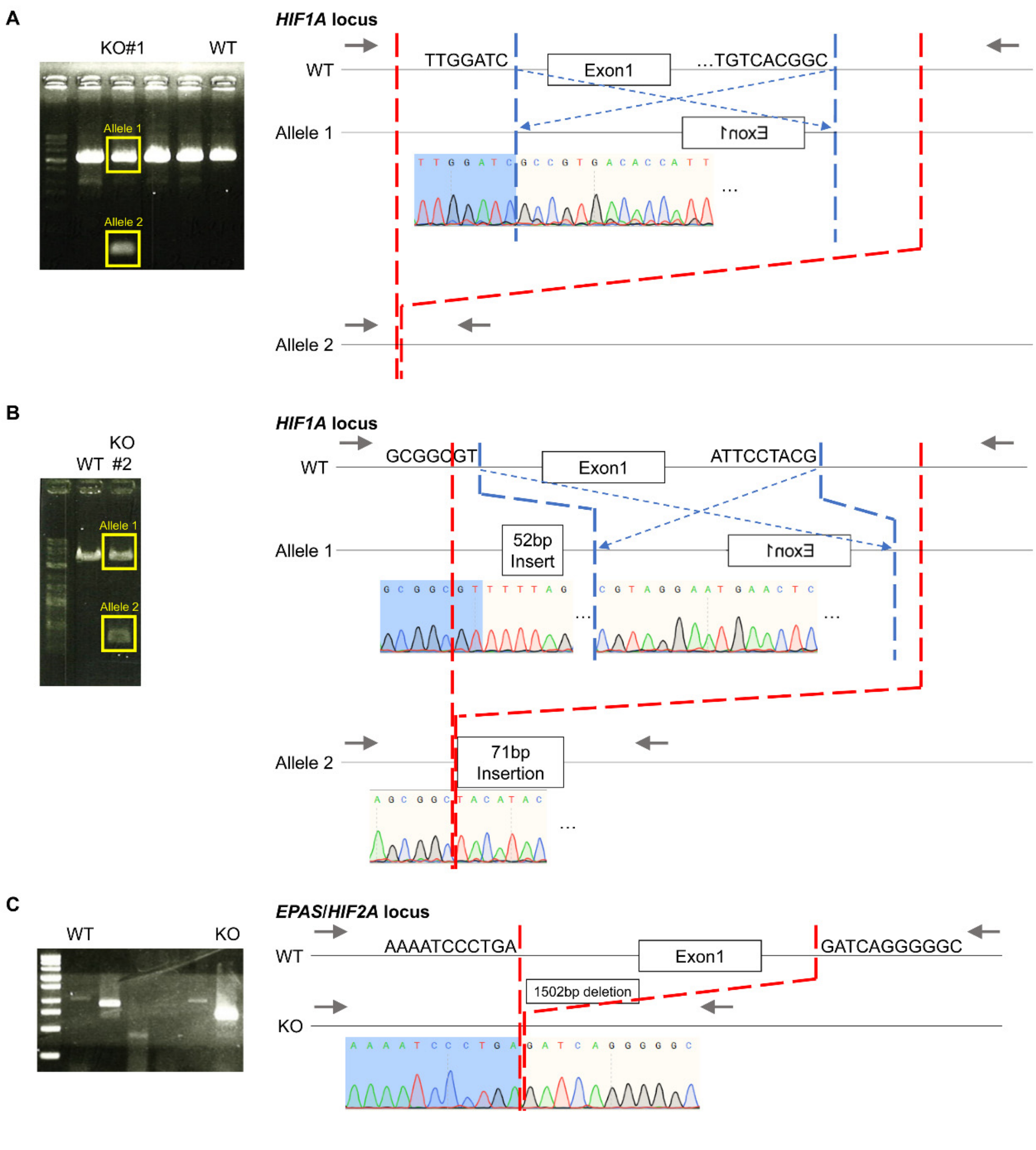

Supplementary Figure S3

A,B. PCR using primers (gray arrows) flanking Exon 1 of the *HIF1A* gene to validate HIF-1α knockout. Genomic sequences of parent HeLa cells and HeLa HIF-1α KO clones (#1 and #2) are as shown.

C. PCR using primers (gray arrows) flanking Exon 1 of the *HIF2A* gene to validate HIF-2α knockout. Genomic sequences of parent HeLa cells and HeLa HIF-2α KO clone is as shown.

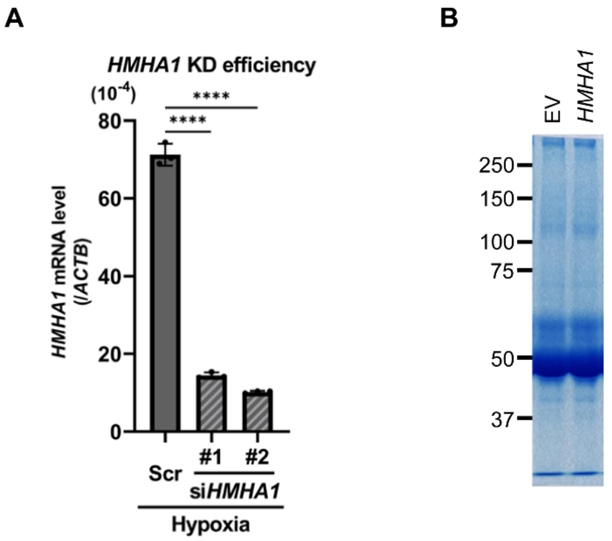

Supplementary Figure S4

A. Efficiency of HMHA1 knockdown with siRNA was verified by qPCR. Results are shown as mean ± s.d. (n = 3); **** $P < 0.0001$ (Student's t test).

B. Coomassie brilliant blue staining after electrophoresis of conditioned medium from HT1080 cells transiently transfected with HMHA1 overexpression vector or the corresponding empty vector (EV) prepared as in the gelatin zymography assay (Fig 4H).

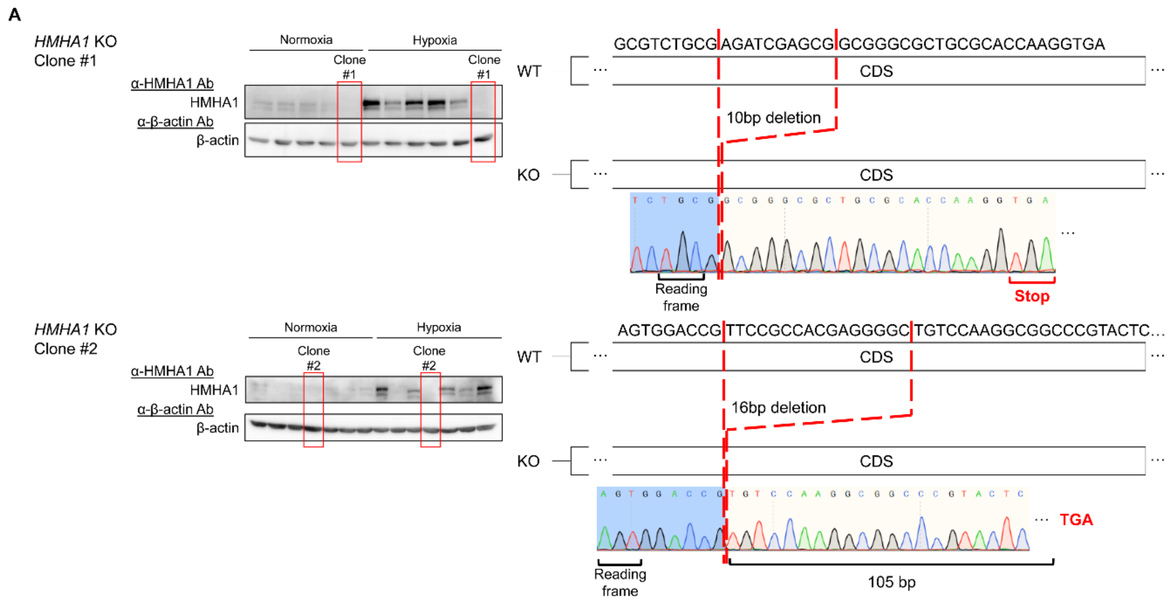

Supplementary Figure S5

A. Western blotting (left) for the indicated proteins to screen for potential HeLa HMHA1 KO clones, followed by genome sequencing (right) for validation.
