# Supplementary material for: Hypoxia- and Postirradiation reoxygenation-induced HMHA1/ARHGAP45 expression contributes to cancer cell invasion in a HIF-dependent manner
Source: Br J Cancer. 2024 May 13;131(1):37–48. doi: 10.1038/s41416-024-02691-x (PMC11231347; doi:10.1038/s41416-024-02691-x)
Supplement: Supplementary file 5 — Supplementary Table S4 [file 41416_2024_2691_MOESM5_ESM.docx]

Supplementary Table S4

| Primers | Sequences |
| --- | --- |
| ACTB Forward | 5’-TGGCACCCAGCACAATGAA-3’ |
| ACTB Reverse | 5’-CTAAGTCATAGTCCGCCTAGAAGC-3’ |
| HMHA1/ARHGAP45 Forward | 5’-GTGCAACAGCTACGTCTACTTCCAG-3’ |
| HMHA1/ARHGAP45 Reverse | 5’-TGTATGGCCAGCGTCTCCAG-3’ |
| HIF1A Forward | 5’-TCA TCCAAGAAGCCCTAACGTG-3’ |
| HIF1A Reverse | 5’-TTTCGCTTTCTCTGAGCATTCTG-3’ |
| HIF2A/EPAS1 Forward | 5’-CACTGCAGACTTGTCCAGTGCTC-3’ |
| HIF2A/EPAS1 Reverse | 5’-CACTGCTCGGATTGTCACACCTA-3’ |
| ARNT1 (HIF-1β) Forward | 5’-CTACCCGCTCAGGCTTTTC-3’ |
| ARNT1 (HIF-1β) Reverse | 5’-CACCAAACTGGGAAGTACGAG-3’ |
| CA9 Forward | 5’-ACCAGACAGTGATGCTGAGTGCTAA-3’ |
| CA9 Reverse | 5’-TCAGCTGTAGCCGAGAGTCACC-3’ |
| VEGFA Forward | 5’-TCACAGGTACAGGGATGAGGACAC-3’ |
| VEGFA Reverse | 5’-CAAAGCACAGCAATGTCCTGAAG-3’ |
| HIF1A locus Forward (PCR) | 5’-CTCTTTCCTCCGCCGCTAAA-3’ |
| HIF1A locus Reverse (PCR) | 5’-CCCCTCAGCTTTCTATACACCAC-3’ |
| HIF2A/EPAS1 locus Forward (PCR) | 5’-CCAGTACCAAATCACCCCGT-3’ |
| HIF2A/EPAS1 locus Reverse (PCR) | 5’-GGGTTCTCCAACCTTCGGTG-3’ |
| CA9 promoter Forward | 5’-TCTCGTTTCCAATGCACGTACAGC-3’ |
| CA9 promoter Reverse | 5’-AGTGACAGCAGCAGTTGCACAGT-3’ |
| 5’ upstream Forward | 5’-ATTCATAGGACACAGGTGTG-3’ |
| 5’ upstream Reverse | 5’-ATTCCTGAGTCCTGACCTC-3’ |
| Intron 1 Region 1 Forward | 5’-TAGGAGGCTGGGATCTGTATG-3’ |
| Intron 1 Region 1 Reverse | 5’-AAGCTGCATTCCAGTGACTC-3’ |
| Intron 1 Region 2 Forward | 5’-AGTCACTGGAATGCAGCTTG-3’ |
| Intron 1 Region 2 Reverse | 5’-CGTCCAGGAAGTCAGAACAATC-3’ |
| Intron 1 Region 3 Forward | 5’-GCTTGCCTTCAGGGAAAAG-3’ |
| Intron 1 Region 3 Reverse | 5’-TGACTCTCACTCCCGGAC-3’ |
